# Efficiency Evaluation of Neuroprotection for Therapeutic Hypothermia to Neonatal Hypoxic-Ischemic Encephalopathy

**DOI:** 10.3389/fnins.2021.668909

**Published:** 2021-09-28

**Authors:** Bowen Weng, Chongbing Yan, Yihuan Chen, Xiaohui Gong, Cheng Cai

**Affiliations:** Department of Neonatology, Shanghai Children’s Hospital, Shanghai Jiao Tong University, Shanghai, China

**Keywords:** therapeutic hypothermia, neonates, hypoxic-ischemic encephalopathy, safety, neuroprotection

## Abstract

**Background:** To evaluate the safety and neurological outcomes of therapeutic hypothermia to neonatal hypoxic-ischemic encephalopathy (HIE).

**Materials and Methods:** Medical records of 61 neonates with moderate to severe HIE were retrospectively enrolled and divided into a therapeutic hypothermia group (*n* = 36) and conventional therapy group (*n* = 25).

**Results:** No significant difference in the incidence of severe adverse events was found between the two groups. Minimum and maximum voltages of amplitude-integrated electroencephalography (aEEG) recording results showed statistically significant differences in therapeutic hypothermia group after 72 h. The neonatal behavioral neurological assessment (NBNA) on the 28th day after birth and Bayley Scales of Infant Development, second edition (BSID II) scores at 18 months old were significant higher in the therapeutic hypothermia group than the conventional therapy group.

**Conclusion:** Therapeutic hypothermia for neonates with moderate to severe HIE improved the development of the nervous system in 0–18-month-old infants and showed a predominant role in reducing death and major neuron development-associated disabilities.

## Introduction

Neonatal hypoxic-ischemic encephalopathy (HIE), mainly caused by perinatal asphyxia, commonly leads to brain damage which manifests as growth disorders, neuro-disability, and even death (Millar et al., 2017). The incidence of brain damage related to perinatal asphyxia (PA) was estimated to be 8.5/1000 live births worldwide, with no recent improvement even in developed countries (Locci et al., 2020). Despite the rapid development of perinatal medicine and neonatal resuscitation techniques being observed in the last few decades, HIE is still an important etiology of neurodevelopmental defects in childhood cerebral palsy, intellectual disability, and epilepsy (Laptook et al., 2017). There is only one proven effective treatment of HIE, therapeutic hypothermia.

Recently, a number of clinical trials and meta-analyses have indicated that therapeutic hypothermia confers benefits in HIE with reductions in mortality and adverse developmental outcomes in full-term newborns with moderate or severe HIE (Silveira and Procianoy, 2015). It has been documented that therapeutic hypothermia for HIE is safe, and can significantly improve the prognosis of the nervous system in children with HIE (Wu et al., 2017). During the treatment of therapeutic hypothermia to neonates with HIE, amplitude-integrated electroencephalography (aEEG) has been used to monitor and evaluate cerebral activity (Thoresen et al., 2010; Skranes et al., 2017). In addition, quite a few studies have investigated the association between aEEG patterns after therapeutic hypothermia and evaluation of neurodevelopment outcome such as Bayley Scales of Infant Development, second edition (BSID II). However, many hospitals in developing countries cannot provide therapeutic hypothermia for neonates with HIE. In the meantime, few studies have explored the relationship between aEEG data before and after treatment with neurodevelopment prognosis. Here, we report the results of therapeutic hypothermia for moderate to severe HIE, and follow-up neurodevelopmental outcomes at the 28th day after birth and at 18 months old to evaluate its safety and efficacy. Moreover, we explore the correlation of aEEG data and neurodevelopment prognosis. We aim to determine the effect of therapeutic hypothermia on neurological outcomes and the prognostic prediction value of aEEG in infants who survive HIE.

## Materials and Methods

### Patients

The Shanghai Children’s Hospital Institutional Review Board approved this study. We performed a single-center, retrospective study of neonates admitted to the NICU of Shanghai Children’s Hospital who were diagnosed with moderate to severe HIE (the diagnosis criteria are described in the Supplementary Material) from January 2010 to December 2016. All parents of HIE patients provided written informed consent, as stipulated by the ethics commit before enrollment. According to the time when our department bought the equipment and started to implement therapeutic hypothermia, infants were divided into a conventional therapy group and therapeutic hypothermia group. Therapeutic hypothermia was approved by the Hospital Ethics Committee and we were required to inform parents and ask them to sign informed consent.

Clinical variables were defined as follows. HIE is a kind of neonatal encephalopathy due to acute hypoxia-ischemia when one or more of following conditions are present. Neonatal signs (as described in the Supplementary Material) consistent with an acute peripartum or intrapartum hypoxic-ischemic event: (1) Apgar score of <5 at 5 min and 10 min; (2) fetal umbilical artery pH < 7.0, or base deficit ≥ 12 mmol/L, or both; (3) acute brain injury seen on brain magnetic resonance imaging (MRI) or magnetic resonance spectroscopy (MRS) consistent with hypoxia-ischemia; (4) presence of multisystem organ failure consistent with HIE (D’Alton et al., 2014). The eligibility criteria for therapeutic hypothermia were as follows: (1) gestational age ≥ 35 weeks and birth weight ≥ 1800 g; (2) Apgar score ≤ 3 at first min, or Apgar score ≤ 5 at fifth min; (3) infants had a pH ≤ 7.0 or base deficit ≥ 16 mmol/L in the umbilical cord or arterial blood during the first hour after birth; (4) infants who had clinical manifestations consistent with HIE or aEEG evidence of abnormal brain function monitoring (Celik et al., 2015). The main criteria for exclusion from the study included; initial normal aEEG monitoring; serious congenital malformations, such as complex cyanotic congenital heart disease (CHD); traumatic brain injury or moderate to severe intracranial hemorrhage; systemic congenital viral or bacterial infection; spontaneous bleeding tendency or platelets less than 50^∗^10^9^/L 12 h after birth (Sarafidis et al., 2014).

### Treatment

The infants in the therapeutic hypothermia group underwent systemic therapeutic hypothermia (Blanketrol^®^ III temperature controlled instrument, CSZ, United States) to maintain rectal temperature at 33.5 ± 0.5°C within 10 h after birth. After 72 h of treatment, infants were rewarmed to rectal temperature of 37°C ± 0.5°C over 6–12 h (<0.5°C per hour). Temperature, respiration, heart rate, blood glucose, blood gas analysis, and electrolytes were closely monitored during therapeutic hypothermia. Once serious complications occurred (as described in the Supplementary Material), therapeutic hypothermia would be discontinued and patients were treated accordingly.

The rectal temperature of infants in the conventional therapy group were maintained at 36.0 ∼ 37.5°C. Meanwhile, the groups both received symptomatic and supportive treatment if needed, such as seizure control, respiratory support, and nutritional support, etc.

### Observation Index

The aEEG was recorded before and after 72 h of treatment for both groups. The neonatal behavioral neurological assessment (NBNA) score on the 28th day after birth and Bayley Scales of Infant Development, second edition (BSID II) followed up to 18 months old were assessed.

Early evaluation criteria of NBNA contain three characteristics: (1) scoring criteria: a total of 20 basic items, divided into 5 parts. Behavior abilities (6 items), passive muscle tension (4 items), active muscle tension (4 items), primitive reflex (3 items), and general evaluation (3 items). Score points have three degrees: 0 points, 1 point, 2 points, out of 40 points; the points method is based on the above 20 items with the choice of gurgling, red ball, talking face response, visual, and other sensibilities, and head erection and support with a total of five items, each score has three degrees: 0 points, 1 point, 2 points, with a full score of 10 points. (2) Criteria: NBNA basic project score < 35 points, suggesting the possibility of brain damage. (3) NBNA scores were performed on 98 subjects on the 28th day after birth, and were trained by specially trained professionals.

For BSID II assessment, a mean (±SD) score of 100 ± 15 was normal on the Mental Development Index (MDI) and Psychomotor Developmental Index (PDI). Severe disability was defined as any of the following: a Bayley MDI score more than 2 SD below the mean score (i.e., below 70), a Gross Motor Function Classification System (GMFCS) grade level of 3 to 5, hearing impairment requiring hearing aids, or blindness. Moderate disability was defined as MDI score 1 to 2 SD below the mean score (i.e., 70 to 84) and any of the following: a GMFCS level of 2, a hearing impairment with no amplification, or an active seizure disorder. Meanwhile the adverse effects of therapeutic hypothermia, severe disability and death were observed at the same time.

### Statistical Analysis

SPSS 17.0 was employed for statistical analysis. The data of normal distribution were expressed as mean ± standard deviation (x̄ ± s). The data were analyzed with *t*-test for differences between the two groups, and with chi-square test for the comparison of the sample rate. *P* < 0.05 was considered statistically significant.

## Results

### General Data

A total of 61 newborns diagnosed with moderate to severe HIE were enrolled from January 2010 to December 2016 in the NICU of Shanghai Children’s Hospital, including 36 cases in the therapeutic hypothermia group and 25 cases in the conventional therapy group.

The baseline characteristics of the two groups are shown in Table 1. There was no statistically significant difference regarding gestational age, birth weight, Apgar score (including 1 and 5 min), gender ratio, cesarean section rate, meconium-stained amniotic fluid (MSAF), and blood pH after birth for the first time (*p* > 0.05).

**TABLE 1:** Comparison of characteristics of 61 neonates with moderate to severe HIE in the two groups.

Items	Cases	Gestational age (week)	Birth weight (kg)	Apgar (mean)		Male ratio (%)	Cesarean section rate (%)	Meconium-stained amniotic fluid (%)	Blood PH after birth
								
				1 min	5 min				
Therapeutic hypothermia group	36	39.4 ± 3.06	3.412 ± 0.427	2.3 ± 1.1	4.2 ± 0.8	23 (63.9%)	15 (41.7%)	8 (22.2%)	7.18 ± 0.32
Conventional therapy group	25	38.39 ± 2.16	3.206 ± 0.368	2.1 ± 1.2	4.5 ± 0.6	14 (56.0%)	11 (44.0%)	4 (16.0%)	7.26 ± 0.15
t/χ^2^		0.142	1.935	0.268	0.351	0.385	0.383	0.361	0.287
*p*		0.8876	0.0577	0.7892	0.7268	0.5324	0.5323	0.5543	0.7751

### Complications

Complications during the 72-h treatment and rewarming period for the therapeutic hypothermia group were recorded and compared with the conventional therapy group. There was no statistically significant difference in cold injury syndrome [0 (0.0%) vs. 0 (0.0%)], thrombocytopenia [5 (13.9%) vs. 4 (16.0%)], infection [7 (19.4%) vs. 5 (20.0%)], electrolyte disorders including: hypokalemia, hyperkalemia, hyponatremia, and hypocalcemia [14 (38.9%) vs. 10 (40.0%)], blood glucose disorder including: hypoglycemia and hyperglycemia [10 (27.8%) vs. 7 (28.0%)], and abnormal liver and kidney function [8 (22.2%) vs. 6 (24.0%)] between the therapeutic hypothermia group and conventional therapy group (Table 2).

**TABLE 2:** Comparison of complications in the two groups.

Items	Cases	Cold injury syndrome	Thrombocytopenia	Infection	Electrolyte disorders	Blood glucose disorders	Abnormal liver and kidney function
Therapeutic hypothermia group	36	0	5	7	14	10	8
Conventional therapy group	25	0	4	5	10	7	6
χ^2^		0.000	0.053	0.003	0.008	0.000	0.026
*p*		>0.05	>0.05	>0.05	>0.05	>0.05	>0.05

### aEEG

There was no statistically significant difference in aEEG recording between the therapeutic hypothermia group and conventional therapy group before treatment [maximum voltage (μV): (18.6 ± 2.5) vs. (22.4 ± 3.1), minimum voltage (μV): (6.5 ± 1.9) vs. (8.2 ± 2.6)]. However, there were significant differences in aEEG recording after 72 h of treatment [maximum voltage (μV): (30.6 ± 2.8) vs. (24.1 ± 3.2), minimum voltage (μV): (13.3 ± 2.2) vs. (9.7 ± 3.4), t = 6.376, 4.257, *p* < 0.05] (Table 3).

**TABLE 3:** Comparison of amplitude of aEEG in the two groups (mean, μV).

Items	Cases	aEEG before therapy		aEEG after 72-h therapy		*t*	*p*
		Maximum voltage	Minimum voltage	Maximum voltage	Minimum voltage		
Therapeutic hypothermia group	36	18.6 ± 2.5	6.5 ± 1.9	30.6 ± 2.8^*ab*^	13.3 ± 2.2^*ab*^	8.951	0.0000
Conventional therapy group	25	22.4 ± 3.1	8.2 ± 2.6	24.1 ± 3.2	9.7 ± 3.4	0.683	0.4975
*t*		1.264	0.852	6.376	4.257		
*p*		0.2111	0.3976	0.0001	0.0001		

*Compared with the conventional therapy group, ^*a*^*p* < 0.05; compared with the aEEG recording before therapy, ^*b*^*p* < 0.05.*

### Comparison of NBNA on the 28th Day and BSID II at 18 Months Old (Follow-Up Study)

The NBNA score of infants who were treated with therapeutic hypothermia was significantly increased compared with the infants in the conventional therapy group (39.1 ± 1.6 vs. 34.2 ± 2.1). The neurobehavioral development outcome was evaluated and followed up to 18 months old by standard BSID II. The MDI in the two groups were 96.5 ± 13.1 vs. 85.2 ± 10.7 respectively. The PDI in the two groups were 98.4 ± 15.7 vs. 86.3 ± 14.2, respectively. As can be seen from the above results, the MDI and PDI of infants in the therapeutic hypothermia group were significantly higher than that of the conventional therapy group (Table 4). Furthermore, severe disability [2 (5.56%) vs. 6 (24.0%)] and death cases [0 (0.0%) vs. 4 (16.0%)] in the therapeutic hypothermia group decreased significantly compared with the infants in the conventional therapy group (Figure 2).

**TABLE 4:** Comparison of NBNA on the 28th day after birth and BSID II at 18 months old in the two groups.

Items	Cases	28th day NBNA score (point)	18 months old BSID II score (point)		Cases of severe disability *n* (%)	Cases of deaths *n* (%)
			MDI	PDI		
Therapeutic hypothermia group	36	39.1 ± 1.6	96.5 ± 13.1	98.4 ± 15.7	2(5.56%)	0 (0.0%)
Conventional therapy group	25	34.2 ± 2.1	85.2 ± 10.7	86.3 ± 14.2	6 (24.0%)	4 (16.0%)
*t*/χ^2^		3.361	7.839	8.573	4.405	6.164
*p*		0.0014	0.0001	0.0000	0.0210	0.0074

### Analysis of Correlation Between aEEG Recording After Therapeutic Hypothermia and BSID II Assessment at 18 Months

**FIGURE 1:**
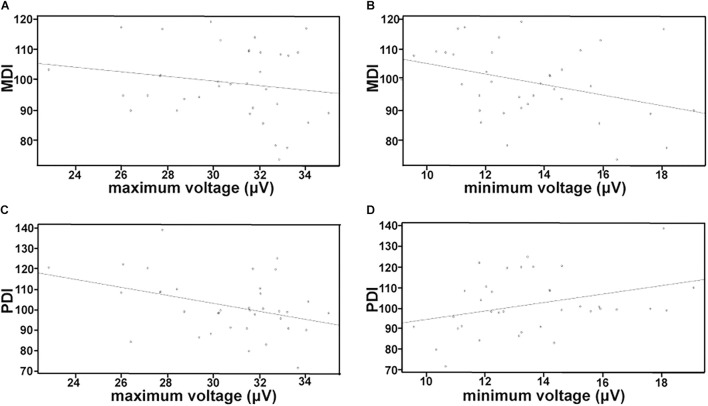
Analysis of correlation between aEEG data and BSID II scores in HIE neonates treated with therapeutic hypothermia. MDI, mental development index; PDI, psychomotor developmental index. **(A)** Correlation between maximum voltage and MDI. **(B)** Correlation between minimum voltage and MDI. **(C)** Correlation between maximum voltage and PDI. **(D)** Correlation between minimum voltage and PDI. The correlation between aEEG recording of HIE infants after therapeutic hypothermia and BSID II scores at 18 months old. There was no significant relationship between maximum voltage and MDI **(A)**, while the minimum voltage record was negatively associated with MDI **(B)**. Likewise, **(C)** shows the negative relationship between the maximum voltage and PDI, whereas the minimum voltage was positively associated with PDI **(D)**.

The correlation between aEEG recording of HIE infants after therapeutic hypothermia and BSID II scores at 18 months old is shown in Figure 1. There was no significant correlation between maximum voltage and MDI (*r* = −0.1763, *p* = 0.3036) (Figure 1A), while the minimum voltage record showed a negative correlation with MDI (*r* = −0.3474, *p* = 0.0379) (Figure 1B). Likewise, Figure 1C shows the negative correlation between the maximum voltage and PDI (*r* = −0.3804, *P* = 0.0221), whereas the minimum voltage had a positive correlation with PDI (*r* = 0.3426, *p* = 0.0408) (Figure 1D). The correlation between aEEG recording after conventional therapy and BSID II assessment at 18 months is shown in Supplementary Figure 1.

**FIGURE 2:**
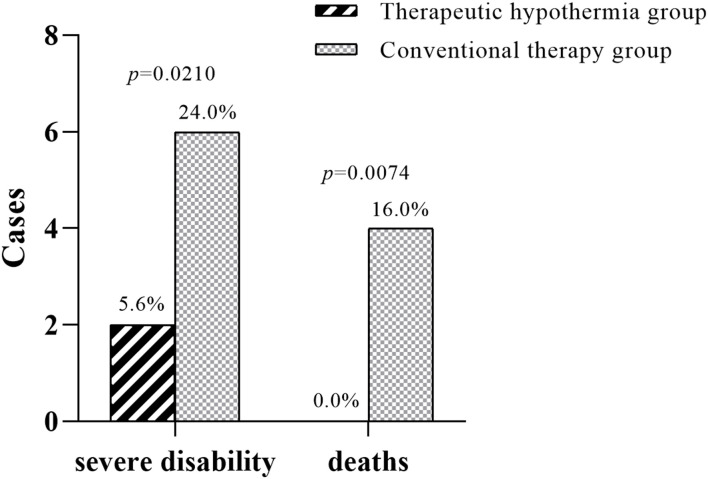
Severe disability and death cases in the two groups. Compared with the infants in the conventional therapy group, severe disability [6 (24.0%) vs. 2 (5.56%), *p* = 0.0210] and death cases [4 (16.0%) vs. 0 (0.0%), *p* = 0.0074] in the therapeutic hypothermia group decreased significantly.

## Discussion

Hypoxic-ischemic encephalopathy is characterized as brain damage after encountering various neonatal hypoxic-ischemic injuries (Millar et al., 2017). Most cases of HIE are associated with risk factors arising before the onset of labor including maternal factors, placental conditions, and fetal problems. It is not only a serious threat to newborns, but also extends into childhood (Kale et al., 2016). Since the 1990s, multicenter randomized controlled studies of therapeutic hypothermia for neonatal HIE were confirmed (Chalak et al., 2014), therapeutic hypothermia has been proven to be safe, reliable, and have a curative effect, and can significantly reduce the neurological sequelae and degree of disability caused by HIE (Jacobs et al., 2013; Pauliah et al., 2013).

Previous studies have reported several serious adverse events during therapeutic hypothermia, including apnea, coagulation disorder dysfunction, serious infection, hemorrhage or thrombosis, and arrhythmia (Kurinczuk et al., 2010). Whereas in our study, very few neonates with HIE developed these complications during therapeutic hypothermia. The outcomes of this study revealed that no infant developed cold injury syndrome in the therapeutic hypothermia group. There was no significant difference in adverse events between the two groups (*p* > 0.05). The results suggested that therapeutic hypothermia was safe for newborns with moderate to severe neonatal HIE under the condition of closely monitoring and performing necessary laboratory tests during the treatment. Therapeutic hypothermia not only significantly decreased the incidence of serious disability and mortality, but also made nervous system prognosis better.

Compared with the conventional EEG, aEEG has the advantages of intuitive graphics, easy analysis, easy operation, and small levels of interference (Schwindt et al., 2015). The results of this study showed that after therapeutic hypothermia of infants with HIE, the maximum voltage and the minimum voltage of aEEG were higher than the conventional therapy group, the increased voltage indicated the amplitude of EEG increased and cerebral activity recovered, which reflected the neuroprotective effect of therapeutic hypothermia (Tamussino et al., 2016).

A concern with any therapy that reduces mortality among infants at high risk of death and disability is the possibility of an increase in the number of infants who survive with disabilities. In our study there was no evidence to show that the moderate or severe disability rates increased at 18 to 22 months old among infants treated with therapeutic hypothermia.

At present, it is emphasized that therapeutic hypothermia must be initialized within 6 h after birth, even during neonatal transport (Akula et al., 2012). Studies have reported that there were significant correlations between the severity of secondary energy failure and the long-term neurological outcome of infants. Furthermore, infants with HIE in the absence of recovery had poorer prognosis (Kim et al., 2013). Therefore, starting therapeutic hypothermia before the secondary energy failure occurs could block HIE caused by hypoxia ischemia, and reduce brain damage (Tang et al., 2016). Therapeutic hypothermia can be divided into induction, maintenance, and recovery temperature phases. An animal experimental study confirmed that therapeutic hypothermia for 72 h has the most significant cranial nerve protective effect, while therapeutic hypothermia for less than 48 h has no obvious effect, and therapeutic hypothermia for more than 72 h has significantly increased complications (Groenendaal et al., 2013). In this study, therapeutic hypothermia was started within 6 h and persisted to 72 h. Our results indicated that severe disability and death cases in the therapeutic hypothermia group were decreased significantly compared with the newborns in the conventional therapy group. MDI was used to evaluate the infants’ acuity of perceptual, discrimination, and response ability. And PDI was used to evaluate the ability of body control and balance, gross motor function, and fine motor manipulation skills. In our study, the NBNA score of the 28th day after birth, MDI, and PDI at 18 months old of infants with HIE in the therapeutic hypothermia group were also improved (Table 4).

Brain development and injury can be evaluated from different perspectives which include a clinical individual perspective (NBNA, MDI, and PDI, etc.) and cell electrophysiological perspective (aEEG, EEG, etc.). Previous studies have shown that aEEG monitoring of neonates provided useful information to evaluate cerebral activity (Finn et al., 2017). The aEEG data provide basic information such as evaluation of neonatal brain development and abnormal brain electrical activity after brain injury. The voltage indicates the intensity of basic electrical activity. With the increase of gestational age, the voltage increases which manifests the continuous establishment of synaptic connections, and the discharge synchronization increases. On the contrary, the voltage decrease indicates the inhibition of electrical activity. Herein, we tried to explore whether data from the aEEG readings of HIE infants after therapeutic hypothermia were correlated with outcome at 18 months old assessed using the BSID-II. We used MDI and PDI scores as separate outcome markers in the 36 HIE infants treated with therapeutic hypothermia. Using Pearson’s correlation analysis, maximum voltage was only negatively correlated with PDI score but not MDI score (Figures 1A,D) in the therapeutic hypothermia group, probably due to its large normal range above 10 μV (al Naqeeb et al., 1999). Whereas the minimum voltage was significantly negatively correlated to MDI and positively correlated to PDI suggesting the subtle balance of these two scores to evaluate neurodevelopment outcome. However, the results were different in the conventional therapy group. It is necessary to expand the sample size in subsequent studies and comprehensively analyze the background, sleep-wake cycle with voltage. Further study on the nervous system prognosis predictive value of aEEG may help for early intervention like rehabilitation exercise, and improve the prognosis of HIE infants.

## Conclusion

In conclusion, our study showed that therapeutic hypothermia with an aEEG monitor is effective and safe for newborns with moderate to severe HIE, and could significantly improve the prognosis according to the BSID II.

## Data Availability Statement

The original contributions presented in the study are included in the article/Supplementary Material, further inquiries can be directed to the corresponding author.

## Ethics Statement

The studies involving human participants were reviewed and approved by the Institutional Review Board, Shanghai Children’s Hospital. Written informed consent to participate in this study was provided by the participants’ legal guardian/next of kin.

## Author Contributions

BW, CC, and CY made substantial contributions to the conception and design, acquisition of data or analysis, and interpretation of data, and involved in drafting the manuscript or revising it critically for important intellectual content. CC and XG revised the manuscript and gave the final approval of the version to be published. All authors agreed to be accountable for all aspects of the work in ensuring that questions related to the accuracy or integrity of any part of the work are appropriately investigated and resolved and read and approved the final manuscript.

## Conflict of Interest

The authors declare that the research was conducted in the absence of any commercial or financial relationships that could be construed as a potential conflict of interest.

## Publisher’s Note

All claims expressed in this article are solely those of the authors and do not necessarily represent those of their affiliated organizations, or those of the publisher, the editors and the reviewers. Any product that may be evaluated in this article, or claim that may be made by its manufacturer, is not guaranteed or endorsed by the publisher.

## Abbreviations

aEEG: amplitude integrated electroencephalography
BSID: Bayley Scales of Infant Development
HIE: hypoxic ischemic encephalopathy
MDI: mental development index
NICU: neonatal intensive care unit
NBNA: neonatal behavioral neurological assessment
PDI: psychomotor development index.
