# Supplementary material for: Efficiency Evaluation of Neuroprotection for Therapeutic Hypothermia to Neonatal Hypoxic-Ischemic Encephalopathy
Source: Front Neurosci. 2021 Sep 28;15:668909. doi: 10.3389/fnins.2021.668909 (PMC8505668; doi:10.3389/fnins.2021.668909)
Supplement: Supplementary file 1 [file Data_Sheet_1.docx]

**Efficiency evaluation of** **neuroprotection for** **therapeutic hypothermia to neonatal hypoxic-ischemic encephalopathy - Supplement**

Bowen Weng, Chongbing Yan, Yihuan Chen, Xiaohui Gong, Cheng Cai^*^

Department of Neonatology, Shanghai Children’s Hospital, Shanghai Jiao Tong University, Shanghai 200062, P.R. China

**Correspondence to*: Cheng Cai, Department of Neonatology, Shanghai Children’s Hospital, Shanghai Jiao Tong University, 355 Luding Road, Shanghai 200062, P.R. China

E-mail: caic@shchildren.com.cn

Running Head: therapeutic hypothermia to HIE

**Methods**

**Patients**

The severity of HIE was assessed mainly according to the modified SARNAT criteria. The moderate stage is defined as: 1) The baby is lethargic with significant hypotonia and diminished deep tendon reflexes; 2) Neonatal reflexes may be sluggish or absent; 3) Apnea; 4) Seizures may occur within the first 24 hours of life. And the severe stage is defined as: 1) Stupor or coma is typical; 2) Breathing may be irregular and the baby often requires ventilator support; 3) Generalised hypotonia and depressed deep tendon reflexes are common; 4) Neonatal reflexes are absent; 5) Disturbances of ocular motion; 6) Pupils may be dilated, fixed or poorly reactive to light; 7) Seizures occur early and often and may be initially resistant to conventional treatments; 8) Irregularities of heart rate and blood pressure are common during the period of reperfusion injury, as is death from cardiorespiratory failure.

The baby with any encephalopathic clinical signs of the following need to be included: 1) hypertonia or hypotonia; 2) disturbance of consciousness; 3) seizures; 4) abnormal postures; 5) neonatal reflexes may be sluggish or absent; 6) apnea or irregular breath; 7) full bregma.

**Treatment**

We commence continuous monitoring the situations of these infant during therapeutic hypothermia to ensure the treatment smoothly. Once the infants had serious complications, such as 1) Life threatening abnormalities of the cardiovascular or respiratory systems; 2) Uncontrolled pulmonary hypertension; 3) Critical bleeding or coagulopathy; 4) So severely affected that there is little hope for normal outcome i.e. moribund or “in extremis” (e.g. very low BP or severe acidosis unresponsive to treatment), therapeutic hypothermia would be discontinued and patients were treated accordingly.

**Supplementary Figure S1**


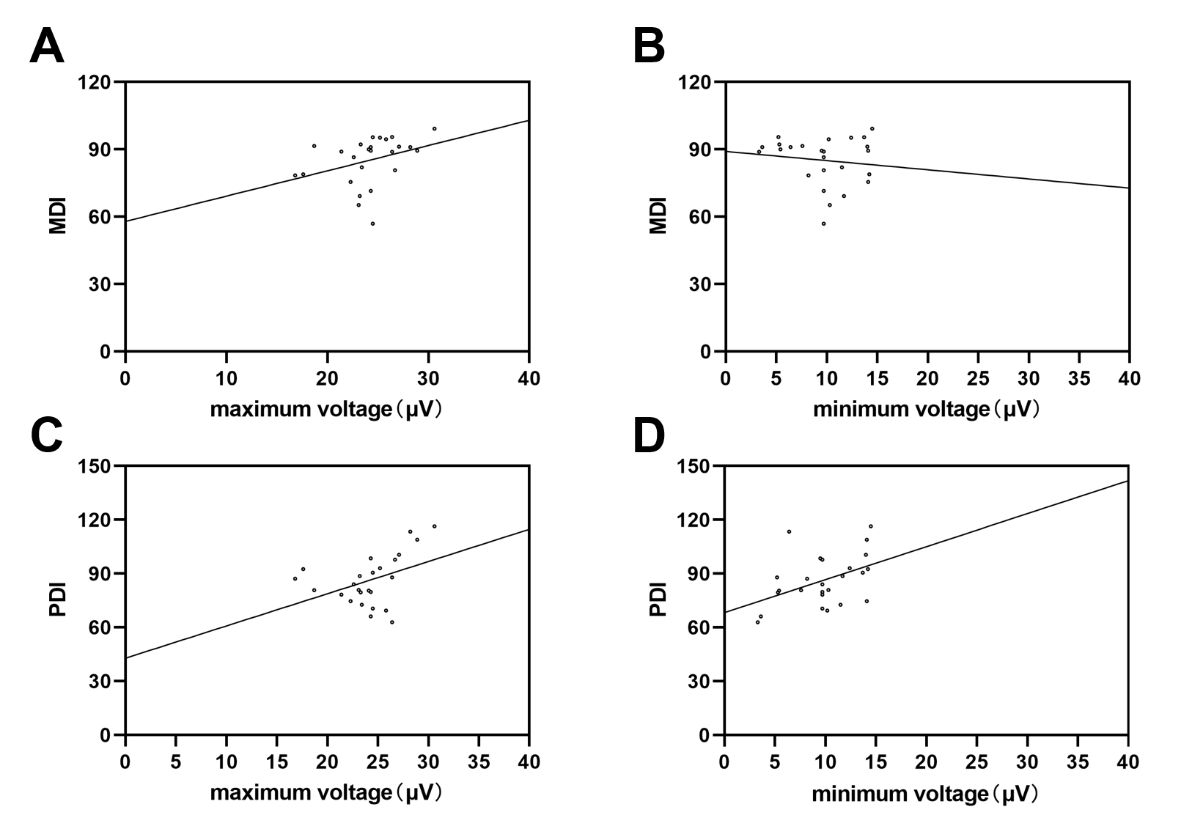


Supplementary Figure S1 legend:

MDI: Mental Development Index; PDI: Psychomotor Developmental Index. The correlation between aEEG recording of HIE infants after conventional therapy and BSID Ⅱ scores at 18 months old. There was no significant relationship between maximum/minimum voltage and MDI (Figure S1A, r=0.3435, *p*=0.0927; S1B, r=-0.1321, *p*=0.5289), while the maximum or minimum voltage were both positively associated with PDI (Figure S1C, r=0.4126, *p*=0.0404; S1D, r=0.4477, *p*=0.0248).
